# Designing Consumer Health Information Technology to Support Biform and Articulation Work: A Qualitative Study of Diet and Nutrition Management as Patient Work

**DOI:** 10.2196/27452

**Published:** 2021-08-10

**Authors:** Courtney C Rogers, Thomas J Moutinho Jr, Xiaoyue Liu, Rupa S Valdez

**Affiliations:** 1 Department of Engineering Systems and Environment University of Virginia Charlottesville, VA United States; 2 Department of Biomedical Engineering University of Virginia Charlottesville, VA United States; 3 School of Nursing University of Virginia Charlottesville, VA United States; 4 Department of Public Health Sciences University of Virginia Charlottesville, VA United States

**Keywords:** Crohn disease, inflammatory bowel diseases, chronic disease, self-management, consumer health information technology, qualitative research, user-centered design, patient work, context, articulation work, diet, nutrition

## Abstract

**Background:**

Diet and nutrition management is an integral component of Crohn disease (CD) management. This type of management is highly variable and individualized and, thus, requires personalized approaches. Consumer health information technology (CHIT) designed to support CD management has typically supported this task as everyday life work and, not necessarily, as illness work. Moreover, CHIT has rarely supported the ways in which diet and nutrition management requires coordination between multiple forms of patient work.

**Objective:**

The purpose of this study was to investigate diet and nutrition management as biform work, identify components of articulation work, and provide guidance on how to design CHIT to support this work.

**Methods:**

We performed a qualitative study in which we recruited participants from CD-related Facebook pages and groups.

**Results:**

Semistructured interviews with 21 individuals showed that diet and nutrition management strategies were highly individualized and variable. Four themes emerged from the data, emphasizing the interactions of diet and nutrition with physical, emotional, information, and technology-enabled management.

**Conclusions:**

This study shows that the extent to which diet and nutrition management is biform work fluctuates over time and that articulation work can be continuous and unplanned. The design guidance specifies the need for patient-facing technologies to support interactions among diet and nutrition and other management activities such as medication intake, stress reduction, and information seeking, as well as to respond to the ways in which diet and nutrition management needs change over time.

## Introduction

Crohn disease (CD), a type of Inflammatory Bowel Disease (IBD), affects approximately 3 million people in the United States [1]. Several factors may contribute to the occurrence of CD, including genetics, environment, and diet [2], but a mechanistic understanding of the disease etiology is unknown [3,4]. CD causes life-disrupting symptoms such as excessive diarrhea, depression, and malnutrition [5] that occur in states of disease remission and inflammatory flare-ups. With no current cure, individuals with CD are tasked with managing their condition, including coordinating complex procedures and regimens [6,7]. As with many chronic conditions, management can include performing multiple tasks and developing skills such as remembering to take medications on time, scheduling appointments, tracking symptoms, seeking social support, and managing nutrition and diet [8].

Though diet and nutrition have not been implicated in causing CD, certain foods can trigger an inflammatory flare-up or exacerbate symptoms for those with CD [9,10]. Moreover, individuals with CD have an increased risk of malnutrition and micronutrient deficiencies [11-14], which are contributing factors to disease morbidity [14-16]. While more research is needed, the current scientific and anecdotal evidence is reason enough for individuals with CD to take diet and nutrition seriously. However, there is no consensus on nutritional or dietary guidelines or a standard nutritional assessment method, making diet- and nutrition-based management challenging [17]. Furthermore, there are limited resources for managing a specific dietary regimen at home [18]. One self-management strategy is to identify and eliminate foods that intensify symptoms [17], typically by adopting an elimination diet and food journaling [19]. Adherence to these methods is demanding, due to social pressures to eat at restaurants, stigma associated with food journaling, difficulty entering reliable dietary information, and difficulty maintaining the habit of journaling [19]. Additionally, stress related to managing cumbersome daily activities, including nutrition management, can contribute to the occurrence of CD symptoms [20] and affect social and emotional well-being [21]. Even without the demands of these activities, adopting an elimination diet may not be successful at mitigating CD symptoms, as there is variability within one’s own metabolism and microbiome over time [4,22]. Therefore, it is increasingly important to develop personalized approaches to diet and nutrition for individuals with CD [23].

Consumer health information technology (CHIT) could address challenges with diet and nutrition management of CD. However, CHIT developed for diet and nutrition purposes only partially addresses the needs of CD management, as features need to be more nuanced to capture the complexities surrounding diet and nutrition. Although tools have been developed for CD, specifically, and IBD, more generally, the apps do not offer robust features for tracking diet and nutrition. Current popular IBD-related apps (eg, GI [Gastrointestinal] Monitor, GI Buddy, and MyCrohnsAndColitisTeam) offer logging capabilities, trend reports, and community forums. These apps lack an integration of features, including those that monitor behaviors and disease states, track diet and nutrition, facilitate connections with providers, develop social networks, provide psychological tools, and provide accurate medical information. As a result, currently available apps lack features to deliver personalized diet and nutrition guidance, integrate this guidance into the broader context of daily CD management, and adapt management activities across a lifespan [24]. Overall, tools tend to treat diet and nutrition management as isolated from other components of management. For CHIT to be a more meaningful part of diet and nutrition management for individuals with CD, its design must be informed by a deeper understanding of these complexities and interactions.

The shortcomings of CHIT designed for CD management may be viewed through the lens of Corbin and Strauss’s [6] theoretical framework of the illness trajectory. In this framework, 3 lines of work are described: (1) illness work (eg, managing medication, scheduling appointments, or tracking symptoms), (2) everyday life work (eg, bathing, eating, or doing laundry), and (3) biographical work (ie, major life events and identity formation). These lines of work often occur in tandem, mutually shape each other, and require coordination known as articulation work [6]. Articulation work is often needed to manage interactions between different tasks [6]; however, CHIT for this condition is often not designed to capture and support interactions between and within the lines of work and, therefore, minimizes the importance of these interactions. Furthermore, CHIT generally supports diet and nutrition management as a generalized, everyday routine, rather than a complex disease management task specific to those living with CD. However, the division between illness work and everyday life work does not necessarily hold for work that has overlapping components such as diet and nutrition management. It is clear that this work is a critical component of illness work, in addition to everyday life work, for individuals living with CD. During times of remission, the overlap between illness and everyday life work may be minimal. However, in times of flare-ups, the overlap could be considerable, with little to no distinction, since every meal requires consideration of the effects it could have on the condition. We refer to patient work [6,25,26] exhibiting these highly overlapping, dual characteristics as biform work. As such, CHIT design for diet and nutrition management for individuals with CD may be understood through the theoretical concepts of biform work and articulation work to explicate and support overlap and interactions, respectively. Therefore, in this study, we aimed to gain a more comprehensive view from a patient perspective of diet and nutrition management through this lens and to provide guidance for how CHIT can be designed to support this work.

## Methods

### Sample

Eligible participants were over 18 years of age, diagnosed with CD, and US residents. Once eligibility was confirmed, a convenience sampling strategy was used to contact individuals to participate in the study.

### Recruitment

**Figure 1:**
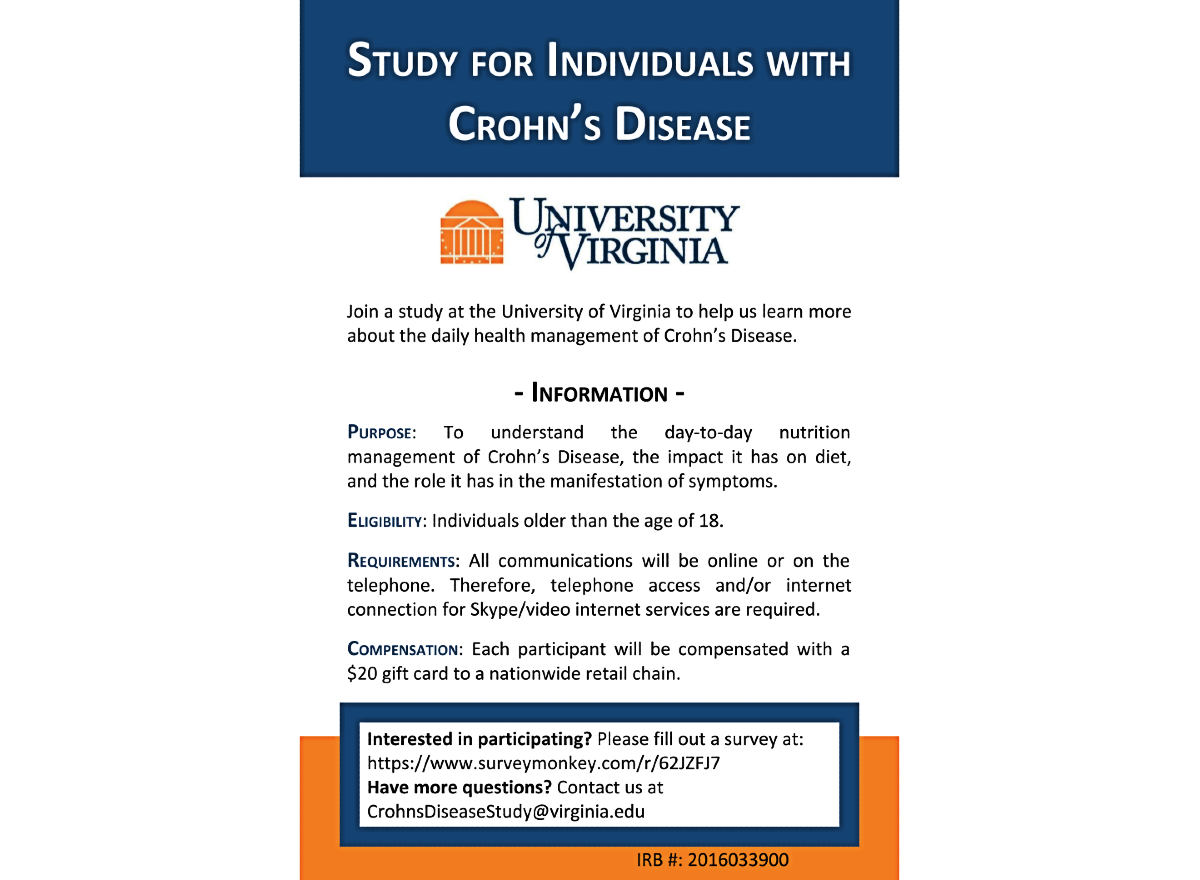
Study recruitment flyer.

Participants were recruited from the online social media platform Facebook [27]. The keyword “Crohn’s disease” was used to search for pages and groups that support individuals with CD. Administrators of both public and private groups were contacted prior to posting information about the study. Posts were directly submitted to pages for approval. Administrators from 9 CD groups and 11 CD pages agreed to the request. We also posted on our personal Facebook profiles. These posts contained a flyer that included information about the study purpose, a link to the recruitment survey on Survey Monkey, and compensation information (Figure 1). The recruitment survey contained questions about basic demographic information (ie, age and gender) and contact information. Compensation was a $20 gift card to a nation-wide retail chain. The recruitment survey was closed after 54 responses were received. Respondents who provided valid information and met eligibility criteria were contacted for an interview. If the respondent did not reply within 24 hours, a follow-up message was sent. Those who did not respond after 2 attempts were not contacted again.

### Data Collection

Data collection took place from October 2016 to February 2018 in 2 phases. Data saturation [28,29] was reached after the second phase of interviews. Interviews were semistructured, lasted approximately 45 minutes, and took place over the phone or on a video-chat platform. We used an interview guide consisting of 15 questions and additional probes. Topics included diet and nutrition management, how diet and nutrition management relate to other aspects of CD management, and experience with existing management tools. The interview guide was not grounded in a particular framework, in order to gain direct information from participants [30]. An informed consent script was read to all participants at the beginning of the interview, and oral consent was audio recorded. Interviews were audio recorded with permission from the participant. Audio recordings were stored in a secured University of Virginia Box account and were deleted from the initial recording device once uploaded.

### Data Analysis

All interview recordings were transcribed. Data were analyzed using QSR NVIVO 11.3, through a conventional content analysis process informed by Hsieh and Shannon [30]. We gained an initial impression of the data and then iteratively drew themes. After analyzing the first 2 transcripts individually, 4 team members determined the preliminary codebook through consensus building [31,32]. The preliminary codebook was reviewed by the senior author (RSV). The next 6 transcripts were divided between the first 3 authors. Then, we came together and decided on the second version of the codebook with input from RSV. The remaining transcripts were analyzed by the first author, and any changes to the codebook were reviewed by RSV. We engaged in simultaneous coding when a participant’s statement was reflective of more than 1 code [31]. This coding framework was used to analyze the remaining data. A final codebook was created to define each theme and subtheme.

### Ethics Approval

This study was approved by the University of Virginia Social and Behavioral Sciences institutional review board.

## Results

### Sample Characteristics

**Figure 2:**
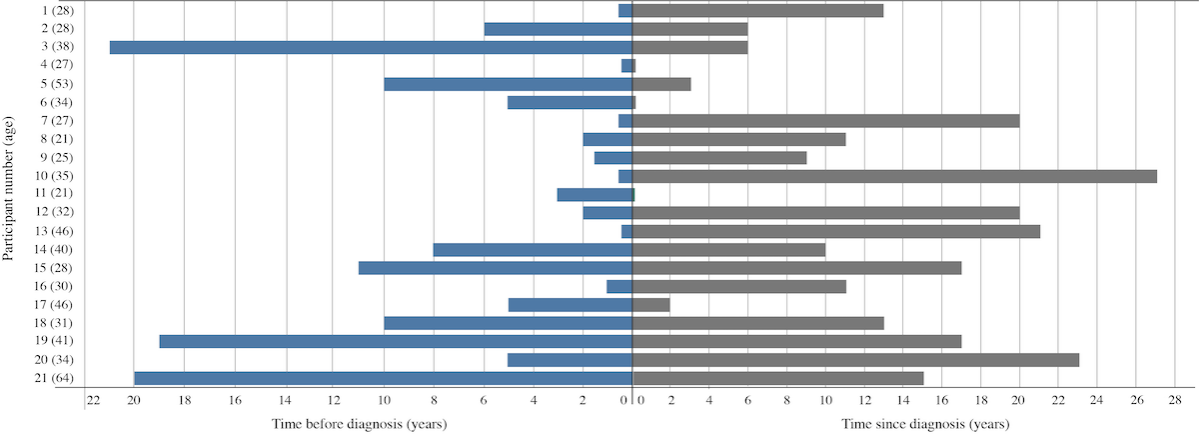
Participant experience with self-management of Crohn disease.

In total, 54 individuals filled out the survey, and, of those, 3 (6%) did not provide sufficient contact information, 27 (50%) did not respond to the follow-up, 3 (6%) were unable to interview due to medical complications, and 21 (39%) were successfully enrolled. Of the 21 participants, 16 (76%) were female, and the average age was 35 years (Table 1). Participants reported living with an official diagnosis of CD for an average of 12 years and felt that they had CD for 6 years, on average, before their official diagnosis (Figure 2).

**Table 1:** Participant demographic data (N = 21).

Characteristic		Participants, n (%)
**Age (years)**		
	20-29	8 (38)
	30-39	7 (33)
	40-49	4 (19)
	50-59	1 (5)
	60-69	1 (5)
**Gender**		
	Male	5 (24)
	Female	16 (76)

### Themes

#### Overview of Themes

Qualitative content analysis yielded 4 overarching themes (Table 2). The themes were oriented around diet and nutrition management as biform work and highlight the articulation work required for CD management. The first 3 themes characterize 3 dimensions of diet and nutrition management, while the fourth theme is orthogonal to the first 3, addressing how tools are used for management.

**Table 2:** Themes and categories identified based on interview analysis.

Theme and subthemes			Definition
**Physical management**			Relationship between diet and nutrition and symptoms of CD^a^
	Management of medication	Relationship between diet and nutrition and prescription drugs	
	Management of remission	Relationship between diet and nutrition and periods with reduced symptoms	
	Management of flare-ups	Relationship between diet and nutrition and periods of increased symptoms	
**Emotional management**			Relationship between diet and nutrition and the psychological aspects of living with CD
	Management of social relationships	Relationship between diet and nutrition and forming and maintaining connections with people	
	Management of routines	Relationship between diet and nutrition and daily tasks	
	Management of stress	Relationship between diet and nutrition and experiences of mental and emotional strain	
**Information management**			Relationship between diet and nutrition and information gathering
	Management of information from health care professionals	Experiences with seeking and obtaining advice about diet and nutrition from trained providers	
	Management of information from text sources	Experiences with seeking and obtaining advice about diet and nutrition from books, websites, and other written materials	
	Management of information from social networks	Experiences with seeking and obtaining advice about diet and nutrition from online and offline connections	
**Technology-enabled management**			Experience using CD management tools for diet and nutrition
	Management experiences using existing tools	Experiences with using technologies to facilitate activities related to diet and nutrition	
	Management needs not met by existing tools	Experiences with lack of usefulness and usability of available technologies related to diet and nutrition	

^a^CD: Crohn disease.

#### Theme 1: Physical Management

Participants discussed the following components of physical management: (1) management of medication, (2) remission, and (3) flare-ups.

##### Management of Medication

Some participants indicated that CD medications positively impact the need for dietary management:

I would say that [the medication] was so good…[that diet] was more of a minor fact that I would forget—not forget that I had a disease, but [I] would live my life more normally.

Participant #7

In other cases, the participants expressed combining medications and dietary management to control and limit flare-ups:

I am on Remicade, and I believe that a diet is very important, and what you eat can try to treat it from the natural side.

Participant #8

One participant with an ostomy bag had trouble with absorbing medications after eating:

[A]bout an hour after I eat, I’m passing whatever through the ostomy—...medication, pills...

Participant #12

##### Management of Remission

When in remission, participants mentioned some consistencies in the foods they tolerate. Participants that committed a restricted diet to memory took several years to develop their diet through trial-and-error processes using food journaling. Many participants did not plan meals and primarily cooked meals at home to control ingredients used. Often, each individual discussed a lack of variation in their diet:

I lead a pretty boring life. I pretty much eat the same thing for breakfast, same thing for lunch. I don’t vary my dinners all that much.

Participant #13

Even with a fixed diet, participants experienced inconsistencies in the foods they could eat:

[Spinach] is fine one day. Then, 2 days later, if I were to eat the same amount of spinach, it’s like, “Oh, holy hell,” my bag [is] blown off. There’s green everywhere, and it’s just a nightmare.

Participant #10

##### Management of Flare-ups

Management activities during remission periods were variable across participants but were more consistent across participants during flare-ups. All participants identified certain foods that tend to cause gastrointestinal distress and increase the risk of a flare-up. The exact type of food varied across participants and for each individual over time. All individuals talked about changing their diet during a flare-up. The majority of participants mentioned eating easily digestible foods such as broths when flare-ups were particularly bad and caused concerns about fluid loss:

Is it gonna be [a flare-up] that runs off of bone broth and crackers, or is it gonna be a full “can’t eat anything and I’m gonna get dehydrated”[one]?

Participant #19

#### Theme 2: Emotional Management

Participants identified multiple dimensions of managing the psychological aspects of living with CD, including management of social relationships, routines, and stress.

##### Management of Social Relationships

Many participants expressed that their unique dietary requirements prevented them from engaging in social activities. An inability to engage in social activities often strained relationships:

When it is bad,…I can’t leave the house, I can’t make plans, I’ve lost friendships with people.

Participant #3

As food is often an integral part of social activities, participants shared feelings of stigmatization when they declined invitations:

The view that I get is probably like, “Oh, she's snobby. She doesn't wanna come out to eat.” I feel that's the stigma they put on me...

Participant #34

If participants did dine out as a social activity, they experienced feelings of sadness in recognizing the foods they can no longer eat. In one case, a participant noted the effect this feeling had on a relationship:

[W]hen I look at a menu, it’s like, “Oh, I can’t have that. I can’t have that.” It’s not a great way of making the other person happy to be around you when you’re depressed that you can’t eat cheese.

Participant #18

##### Management of Routines

Since diet and nutritional management of CD is highly individualized, several participants felt frustrated with using trial-and-error methods to devise routines to follow. Food and eating were sources of tension for participants in their management routines:

I could probably say I am afraid of [food]. It sounds really weird…that you are so afraid of food.

Participant #5

When there were deviations in routine, participants experienced increased difficulties with managing diet and nutrition:

Trying to travel with a diet is very difficult...Everything's set around food...You go to the kid's functions, you go to [visit] other family—anything—everything's food oriented.

Participant #17

Some participants discussed the financial strains of incorporating diet and nutrition into their routines:

I don’t want [my family] to have to go out of [their] way to pay more for food, just so that I can possibly eat it.

Participant #11

##### Management of Stress

Participants stated that increased stress from everyday life exacerbated their symptoms and, in turn, affected their diet and nutrition:

[S]tress is a major trigger with this disease...If it’s emotional, if it’s traumatic, if it’s work related, the stress will affect the disease.

Participant #19

In one case, the uncertain nature of diet and nutrition as well as of the disease in general was a source of daily stress:

I guess just not knowing when the symptoms are gonna pop up—it's very difficult when you're dating or at school or working...Stress definitely triggers it and makes me sicker.

Participant #18

To manage the stress levels and the mental health impacts of CD, several participants sought professional assistance:

I’m in therapy...I’ve always had chronic depression because of the Crohn’s. We go over stress levels and all that stuff.

Participant #12

#### Theme 3: Information Management

Participants reported gathering information from health care professionals, text sources, and social networks.

##### Management of Information from Health Care Professionals

There was a consensus among participants that it is rare to gather useful information about diet and nutrition management of CD from health care professionals. One participant noted about a physician:

The doctor has never even said anything [about nutritional needs]. That would be another very helpful thing for people with CD to find the right food to help their body.

Participant #5

Moreover, some participants were frustrated with the advice given by dieticians, as it was often not CD-specific:

I spoke with a dietician a few years ago, and they literally quoted the [Food and Drug Administration] guidelines. I'm looking at them, [and I’m] like, “I have Crohn's. I thought you were a dietician for Crohn's.”

Participant #17

##### Management of Information From Text Sources

Most participants cited gathering information from internet searches as well as professional and nonprofessional websites:

[I] did a lot of internet researches. There’s the Crohn’s & Colitis Foundation that I’ve got a lot of information on.

Participant #16

Though the internet was the most frequented for information, some participants favored books for diet and nutrition information:

I love books, so I will pick the library first, over the internet.

Participant #19

##### Management of Information from Social Networks

Though participants received and sought information from health care providers and text sources, they discussed information from social networks more at length. Participants noted using anecdotal information from online and offline social network members to identify foods that may or may not contribute to the risk of a flare-up. For instance, a participant explained:

I'm [in] a support group on Facebook and a lot of [them] say, “I don't eat leafy greens, so I couldn't tell you what they do to me.” That's what everybody says.

Participant #14

Participants tended to glean information and support from family members and close friends in their social network. In several cases, family members were involved in managing diet-based decisions, because they had CD, were respected by the participant, or were professionally trained in nutrition. However, they were not the participant’s formal health care provider:

My dad, he also has Crohn's as well as my brother, who also has Crohn's...[W]e can all relate to the issue that we're experiencing.

Participant #18

Information about recipes, meal planning, and living with a restricted diet was also found by participants on social media:

I use Instagram a lot, because a lot of people post things there, especially, recipes or ideas or things that work for them, kind of similar to the forums we talked about earlier.

Participant #3

Social media activity varied among participants, where some were active in posting and others preferred to only read the posts in pages and groups.

#### Theme 4: Technology-Enabled Management

Participants reported using various tools for CD management and described their experiences using these tools, critiquing aspects that were not useful and noting limitations in usability.

##### Management Experience Using Existing Tools

Participants expressed a range of perspectives on available tools. A few participants identified benefits of food diaries immediately after a diagnosis of CD to help figure out an initial effective diet. One noted that:

For 7 or 8 months, I kept a food journal with any symptoms I might be having...[T]hat really helped me know what I can and can’t eat.

Participant #2

Other participants found food diaries time consuming, difficult to keep up with, and, often, hard to gather valuable information from, due to the inconsistent nature of diet and nutrition:

I tried to [track my diet], but I am really bad about that. Even keeping track of that, you still don’t know, because you could eat something and you feel fine with it. And, next time you eat it, you feel terrible.

Participant #1

Although there are tools available for tracking diet and nutritional content, participants reported not using these tools for prolonged periods due to their limitations. One limitation noted by participants was that these available tools were not designed for CD management and, thus, were not perceived as useful:

I probably would [use a tool] if I had something that I liked. And, trust me, if there was something out there that I thought was amazing, I would have found it.

Participant #3

Participants tried using a range of apps such as FitBit, MyFitnessPal, and MyPlate; however, one participant noted:

[I found myself] going back to pencil and paper and writing things down.

Participant #21

This was because of difficulties navigating app interfaces. Overall, participants reported a lack of usefulness and usability of currently available technology designed to track diet and nutrition.

##### Management Needs Not Met by Existing Tools

The cohort interviewed had limited prolonged engagement with CHIT, due, in part, to the lack of CD-specific tools available. However, participants wanted to engage more with CHIT, and they discussed features that integrate various aspects of illness and everyday life work. First, several participants stated that a tool to improve locating and accessing bathrooms would be helpful, particularly, during flare-ups:

I think [it would be helpful] if somebody were to develop an app with the technology...[that] would tell you what gas stations or what stores are nearby...[and] have a public restroom...and how accessible are those bathrooms—a single stall, or is it a multi-stall?

Participant #10

Notably, many of the participants voiced a desire to have better connections with other individuals with CD in their local area:

I actually have been seeking out these support groups. Some Facebook group that I'm on for Crohn's, they give out a roll call, trying to find out where everyone is living...Someone just connected me with—to an in-person support group—...I will likely end up joining that.

Participant #18

Participants were interested in automated features and tracking trends related to behavioral factors such as diet and nutrition and disease states:

A statistical analysis to find out how am I doing—[a]n app—would be great, or, a beautiful spreadsheet just [for] trends, that would be great.

Participant #19

Participants expressed a desire for integrated app features or the ability to synchronize data from multiple apps:

It looks like [recent apps designed for individuals with IBD] track food—...I didn’t really wanna separate [the functions]. That way, if I wanted to look back, it wouldn’t be in 2 places. I've already got My Plate activated with my food for my history now.

Participant #17

More specifically, a few participants stated that they would appreciate connections to health care providers:

I think it would be really great if my doctor was connected to [an app], somehow—[if] the information got sent over,...so they had access to how I'm [doing]...

Participant #20

## Discussion

### Principal Results

Qualitative content analysis revealed 4 themes: physical, emotional, informational, and technology-enabled management. Across participants, diet and nutrition management was an integral part of both illness and everyday life work. However, the extent to which diet and nutrition management could be considered as biform work varied, not only by participant but also over time. Participants usually attempted to manage their diet during remission to prevent flare-ups. For some participants, the use of medication reduced—or, in some cases, eliminated—the need to manage diet and nutrition as illness work. Illness work was also reduced via relying on a consistent diet. During flare-ups, the need for diet and nutrition management as illness work was imperative, as each participant reported maintaining a list of “safe foods” that they rely on. Participants discussed the centrality of stress in CD management, as it tended to exacerbate symptoms, in turn, causing an increased need to manage diet and nutrition as illness work. For many participants, stress stemmed from frustrations with their limited diets and subsequent difficulties developing and maintaining social relationships. As information from professionals was often insufficient, participants relied mainly on social networks to learn about the dietary aspects of CD management. Obtaining and seeking information about diet and nutrition management was often illness work, as participants typically used this information to avoid or ease disease symptoms. Lastly, statements about articulation work were pervasive throughout the interviews. Tasks such as taking medication and eating needed to be coordinated to avoid adverse outcomes. In other cases, tasks were inextricably linked, such as managing stress along with diet and nutrition. For some participants, stress was caused by the diet-restrictive nature of CD, in turn, affecting disease severity and subsequently increasing the need to manage diet and nutrition.

Participants identified the need for complex and integrated functionalities for CHIT to support diet and nutrition management, as there are varying degrees of overlap between illness and everyday life work. Coordination is also required between and within lines of work. The need to manage diet and nutrition as illness work fluctuated over time for participants; however, CHIT rarely responded to oscillations in times of remission and flare-ups, as participants predominantly noted these using general apps designed to track physical activity and calorie expenditures. Moreover, available tools did not support information management as illness work, since participants sought information about diet and nutrition mostly from social networks. For those that used management tools, many reported paper-based food journaling as cumbersome illness work. Additionally, a majority experienced limitations related to usefulness and usability in available tools to meet these needs. Altogether, participants desired features that are more responsive to the realities of living with CD and the interconnected nature of this condition. These desired features included automated diet and nutrition tracking, facilitated social connections, a public restroom finder, analytics, information-sharing with health care professionals, and integration with other apps that are already part of their daily workflow.

### Elaboration on Theoretical Concepts

Even though we initially proposed the concept of biform work, the results from this study show that this concept is not necessarily fixed with regard to diet and nutrition management for people living with CD. There were instances where this type of management was both illness and everyday life work and also instances where it largely became everyday life work. Even if diet and nutrition management existed in a state of everyday life work, psychological factors often could trigger a flare-up for participants and, consequently, increase the illness work–related characteristics. Though not the focus of this study, there may be an opportunity to explore these psychological factors more deeply, as diet and nutrition management could also be a component of biographical work. Thus, this type of management could exist as multiform work, meaning that there could be overlap between everyday life, illness, and biographical work. The concept of diet and nutrition management as multiform work could be meaningful in other conditions such as obesity and anorexia. In such conditions, the need to manage diet and nutrition can be illness and everyday life work, in addition to biographical work, as relationships with food can contribute to self-identity [33-36]. By examining when and how diet and nutrition management can exist as multiple lines of work, a more holistic perspective can be elucidated, and, as a result, interventions can be developed to be more responsive to the multifaceted realities of diet and nutrition management of many chronic conditions.

Since the extent to which diet and nutrition management existed as biform work varied among the participants and over time, the reported need for articulation work between and among other management tasks varied as well. Articulation work was needed for task coordination, aligning with the original conceptualization of this type of work [6]. The understanding of articulation work in the context of chronic disease management has generally remained the same, focusing on organizing, delegating, scheduling, and regimenting consistent tasks [37-41]. However, for the participants in this study, articulation work was not so linear, as management needs typically were in flux and could change instantaneously. Also, management tasks, in some cases, mutually shaped each other and, by their nature, could not be regimented. For example, stress from daily life events triggered increases in symptoms and subsequent increases in management tasks. This kind of stress can be unpredictable and, for example, could stem from an impromptu invitation to a social gathering. As such, there is a need to expand on the understanding of articulation work, so that interventions can better attend to the interconnected nature of diet and nutrition management and other management tasks.

### Comparison With Previous Literature and Implications for Design

Interactions between the first 3 themes yielded several insights into how future patient-facing technologies can be developed to support diet and nutrition management as biform work, when needed, and facilitate articulation work. The fourth theme informs potential features and functionalities of these technologies.

The first theme demonstrated the articulation work needed to manage diet and nutrition and medication. The interconnected nature of these factors is supported by the scientific evidence, as both food and medications have been shown to affect the composition of the gut microbiome [42,43] and, thus, inflammation in the intestine [44]. As early research demonstrates the individualized and variable nature of diet and nutrition’s association with CD [42], general dietary guidelines may not be appropriate, and individualized guidance may need to be developed. Currently, CHIT designed specifically for CD does not support this type of articulation work, as the majority of tools do not make connections between medication and food intake [45,46]. To assist with this work, technology that leverages pattern detection through machine learning [47] could be designed to facilitate the identification of individualized diet and nutrition guidance by capturing and coordinating food and medication intake and monitoring symptoms. Although several participants discussed the difficulties of food journaling, others found that smartphone barcode scanning features, such as the one in the MyFitnessPal app [48], made it easier to track food intake. Moreover, the addition of nutrient content information in food databases within an app could help to mitigate malnutrition by tracking both macronutrients and micronutrients, which has shown to be important in CD management [14,49]. This capability is not readily available in the MyFitnessPal app [11] or other apps with barcode scanning functionality. Incorporating these features into technology could address barriers in capturing food intake [19]. Additionally, machine learning at both the individual and aggregate level could be used to identify both safe and irritating foods and automate meal planning by identifying acceptable ingredients and substitutions, reducing the work required and increasing the utility of technology that facilitates food journaling [9,10]. Lastly, customized features that adapt to individual needs over time could support the fluctuations in diet and nutrition management as biform work. For example, if medication has eliminated the need for an individual to manage diet and nutrition as illness work, a user could then hide the food journaling feature and rely on the symptom tracker to reopen this feature if a flare-up is imminent.

The second theme shows that for the participants in this study, psychological factors also had an effect on the extent to which diet and nutrition needed to be managed as biform work. Participants described how diet and nutrition can affect social well-being and mental health, in turn, affecting disease states and requiring more articulation work. This finding supports the evidence that individuals with food restrictions have reduced social and emotional well-being [21], experience increased levels of stress and anxiety [50], and experience stress that, in turn, affects disease severity [20]. Moreover, this study provides further evidence that the processes of diet and nutrition management, not only disease severity and illness perceptions, are a source of stress [51]. Though a few apps designed to support diet and nutrition management for individuals with CD allow for mood tracking [45,46], these features are still nascent and neither include capabilities to track social engagements nor are adaptive to the extent to which these factors exist as biform work. Patient-facing technology could assist in monitoring and reducing stress and promoting social well-being by supporting biform work when needed and elucidating interactions among diet and nutrition, social engagement, and stress. Mobile sensing can identify increased stress levels [52,53] to facilitate activities [54] such as providing meditation guidance or prompting a connection to a health care provider [55]. By monitoring stress levels, these data can be analyzed along with diet and nutrition, medication, and symptom data. Machine learning could be applied to explore the relationships between these factors to further refine a food irritant profile and identify patterns in flare-ups. This profile could be used to identify restaurants and specific menu items that align with a user’s restrictions, which could reduce the psychological burdens participants expressed they experience when attending social activities. Additionally, these data can be synthesized with location and calendar data, which could help to predict stressful events based on past data related to daily activities and stress. Location and calendar data could also be cross-referenced with data available on public restrooms [56], to automatically have a restroom option available to the user, reducing stress in urgent situations. As some apps have started to integrate behavioral insights [46], these features could be expanded by using predictive analytics to identify when flare-ups are about to occur and provide recommendations for foods that are least likely to initiate or exacerbate a flare-up and preemptively suggest users cancel activities. By tracking a wide range of contextual factors beyond food intake, there is an opportunity to explore why participants experienced inconsistencies with fixed diets. Though there are apps that are providing support to track a broad range of variables, these apps aim to provide data to health care providers to improve clinical care, rather than to support individuals with CD in performing biform and articulation work [46].

As discussed in the third theme, participants sought and obtained diet and nutrition information from health care providers, text sources, and social networks. Similar to this study, one study documented that individuals with IBD wanted to know more about dietary management to reduce flare-ups and noted limitations in dietary advice offered by health care providers [57]. Additionally, seeking health information on social media is increasing in prevalence [58,59]. Available apps offer a range of education materials, and some allow connections to health care providers [60-62]. A few apps offer social networking functions [45,46], which increases the articulation work needed to find and integrate information into disease management strategies. To limit articulation work, technology could help promote online relationships among individuals with CD by suggesting connections based on similar food profiles to share recipes and general diet and nutrition information. Furthermore, those who have similar types of daily routines could be connected to share diet and nutrition management techniques to reduce the illness component of biform work. Additionally, features that connect individuals to health care providers could be expanded to facilitate appointment reminders and scheduling, to promote clinical management, as this has been shown to be a key component of long-term management of CD [63,64]. The collection and sharing of the patient-generated health data with providers could also support clinical care of patients with CD [65-68]. Lastly, the aggregation of these data across users, such as those in the IBD Partners research network [69], can be used to collect the amount of data needed to develop personal nutrition guidance through machine learning [70], deliver precision medicine [71-73], and promote citizen-science in health care [74]. However, it is important to note that there are uncertainties surrounding the amount and variety of data required to build and validate predictive models, as studies range from using the n of 1 study design [75] to including hundreds [76] or thousands of participants [77].

### Limitations

Although this study provided several insights about diet and nutrition management for CD, it also had a few limitations. Since we asked participants to describe CD management over time, the data could be subject to recall bias. However, the participants were not asked to recount certain details about events but, rather, asked to reflect on the most salient points of diet and nutrition management and their overall experience with CD. By recruiting on social media, the sample inherently was comfortable using technology, and, thus, this study did not allow for an understanding of the needs of those with limited technology experience. However, use of this sampling strategy could be beneficial, as the participants could identify challenges with the technology that did not necessarily stem from general inexperience or discomfort using technology. Additionally, apps that partially address some of the needs identified may have been developed since the study was conducted. However, our discussion section acknowledges these apps when they are relevant to a need that was articulated by participants. Lastly, the CHIT guidance provided centers around tracking symptoms to improve outcomes. It has been shown that monitoring biomarkers may be a more effective indicator of disease progression than tracking symptoms alone [64]. Nonetheless, symptom management is an important part of an integrated approach for managing CD and has been shown to be effective in improving the quality of life for individuals with CD [78-80].

### Conclusions and Future Directions

Our interviews with individuals with CD reflected 4 main themes, illustrating the fluctuating biform nature of diet and nutrition management as well as the continuous and, in some cases, spontaneous articulation work needed to manage complex interactions between diet and nutrition and other aspects of life with CD. The participants in this study experienced numerous challenges with diet and nutrition management and use of existing technologies to support management activities. Future work should focus on other chronic conditions that require diet and nutrition management to supplement the understanding of biform work, explore the concept of multiform work, and expand on the definition of articulation work. Methods such as journaling could help to capture the nuances and complexities of daily diet and nutrition management. This study also provided descriptive and prescriptive design guidance [81] for CHIT designed to support the holistic and variable experience of diet and nutrition management for individuals with CD. Subsequent research should focus on using this guidance as a foundation for original designs or to redesign technologies for this purpose. Refinement of the proposed design guidance through an iterative participatory design process is essential for the development of interventions that will benefit individuals with CD and promote long-term engagement [82].

## Abbreviations

CD: Crohn disease
CHIT: consumer health information technology
IBD: inflammatory bowel disease
